# Comparison of temperature rise in the pulp chamber with different light curing units: An *in-vitro* study

**DOI:** 10.4103/0972-0707.71644

**Published:** 2010

**Authors:** A V Rajesh Ebenezar, R Anilkumar, R Indira, S Ramachandran, M R Srinivasan

**Affiliations:** Department of Conservative Dentistry & Endodontics, S.R.M. Dental College, Chennai, India; 1 Department of Conservative Dentistry & Endodontics, Ragas Dental College, Chennai, India; 2 Department of Conservative Dentistry, Venkateshwara Dental College, Chennai, India

**Keywords:** LED units, QTH units, temperature rise

## Abstract

**Aims/Objectives:**

This *in vitro* study was designed to measure and compare the temperature rise in the pulp chamber with different light curing units.

**Materials and Methods:**

The study was done in two settings-*in-vitro* and *in-vivo* simulation. In *in-vitro* setting, 3mm and 6mm acrylic spacers with 4mm tip diameter thermocouple was used and six groups were formed according to the light curing source- 3 Quartz-Tungsten-Halogen (QTH) units and 3 Light-Emitting-Diode (LED) units. For the LED units, three modes of curing like pulse-cure mode, fast mode and ramp mode were used. For *in-vivo* simulation, 12 caries free human third molar tooth with fused root were used. K-type thermocouple with 1 mm tip diameter was used. Occlusal cavity was prepared, etched, rinsed with water and blot dried; bonding agent was applied and incremental curing of composite was done. Thermal emission for each light curing agent was noted.

**Results:**

Temperature rise was very minimal in LED light cure units than in QTH light cure units in both the settings. Temperature rise was minimal at 6mm distance when compared to 3 mm distance. Among the various modes, fast mode produces the less temperature rise. Temperature rise in all the light curing units was well within the normal range of pulpal physiology.

**Conclusion:**

Temperature rise caused due to light curing units does not result in irreversible pulpal damage.

## INTRODUCTION

Temperature rise, during curing of light activated restoratives, relates both to the polymerization exotherm of the material and also to the thermal emission from the dental light curing unit.[1] Zach and Cohen[2] stated that a temperature rise of 5.5°C within the pulp chamber would lead to irreversible pulp damage.[2]

Light curing units can cause a temperature increase that could lead to irreversible pulpal damage.[3,4] The potential damaging effect of temperature increase on pulp tissue during restorative treatment has been a major concern in the field of dentistry for many years. Thermal transfer to pulp is influenced by material shade, thickness, composition, porosity, curing time and residual dentin thickness.[1,5,6] Temperature rise during the curing of light activated restorative materials is, however, mainly contributed by the light source.[7] It also varies with the type of curing unit, quality of light filter, output intensity and irradiation time.[1,8] The heat emitted by the individual curing lights depends on the various curing modes used.[9]

This study was designed to measure (i) the pulpal surface temperatures when using Quartz-Tungsten-Halogen (QTH) and Light Emitting Diode (LED) light source, by quantifying and comparing the thermal emission of these light-curing sources, (ii) temperature changes associated with varying curing modes of each light curing unit *in vitro* and (iii) the simulation of the *in-vivo* situation of a composite restoration, where step-wise preparation and temperature changes were recorded after each step.

## MATERIALS AND METHODS

The materials required were the acrylic base plate and acrylic spacers of 3mm and 6 mm each to measure the thermal emission. The Light Emitting Diode (LED) curing devices used were the Confident LED light curing unit (Group 1), Satellac Mini LED light curing unit (Group 2) and Satellac S.P. LED light curing unit (Group 3). The Confident LED light curing unit and the Satellac Mini LED light curing unit provide three different modes of curing like Pulse cure mode, ramp mode and fast mode. Sub-groups were made for these 3 modes for the above-mentioned LED curing units. Thus, Group 1a denotes pulse cure mode in Confident LED light and Group 1b and 1c denotes ramp mode and fast mode in Confident LED curing light, respectively. Similarly Group 2a denotes pulse cure mode in Satellac Mini LED light curing unit and Group 2b and 2c denotes ramp mode and fast mode in Satellac Mini LED light curing unit, respectively.

The halogen light curing units used in this study were the 3M light curing unit (Group 4), Densply light curing unit (Group 5) and Cu-100A light curing unit (Group 6). A K-type thermocouple with tip diameter of 4 mm and 1mm each secured to the digital thermometer was used to evaluate the thermal emission. A water bath at 37°C ±0.1°C was used to simulate the oral environment.

Thermal emission of each light curing unit was measured by a K-type thermocouple and a digital thermometer. The thermocouple was secured onto a groove in an acrylic base plate so that the surface of the thermocouple was flushed against the top surface of the base plate. Two clear acrylic plates of 3 mm thickness with a 7 mm diameter hole was served as spacers to control the thermocouple light guide exit window distance (refers to the distance between the two spacers, i.e. 7mm, through which the blue light from the curing unit passes and reaches the tip of the thermocouple probe). The thermocouple is positioned at the center of the 7 mm hole. The light guide exit windows of the Light curing units (LCU) were placed over the 7 mm hole of the upper acrylic plates and activated. Temperature rise during irradiation can be measured at distances of 3 mm and 6 mm away from the thermocouple.

The procedure is then repeated similarly with two 6 mm acrylic spacers and the temperature rise was registered. Data was then subjected to one-way ANOVA and Independent samples – *t* – test at significance level 0.05. The mean maximum temperature rise of the different LCU’s / curing modes was compared to the conventional halogen light cure unit. Also, the difference between the curing modes for the same light and different LED lights were compared. Temperature changes at 3 mm and 6 mm were also contrasted.

### *In vivo* simulation of composite resin restoration

To simulate *in vivo* condition, 12 caries free human third molar teeth with fused roots were selected (2 for each light source). A2 Composite shade filling material (Z-100 3M) and 3M etchant (Scothbond Multi-purpose etchant) and bonding agent (3M Single Bond) were used in this study. All the teeth were cleaned with sodium-hypochlorite solution and stored in physiologic saline. To gain access to the pulp chamber, apical 3 mm of the root was resected. The tooth was immersed in a water-bath at 37°C ± 0.1°C. A new thermocouple probe was designed with a smaller diameter (1mm) at the tip. Thermocouple probe was inserted through the apex up to the pulp chamber and the position verified radiographically.

Occlusal cavity was prepared with the following specifications-2 mm width, 5 mm length and 3mm depth. For each light-curing unit, 5 consecutive 40 sec curing exposures were made on the occlusal surface of the prepared tooth and temperature rise registered. Total etching with 30% ortho-phosphoric acid was done next for 20 sec and rinsed with water for 15 sec. After blot drying for 5 sec, bonding agent was applied and cured for 20 sec and temperature rise during this period was registered. Incremental curing of 1mm was done up to 3mm. Each increment was cured for 40 sec and temperature rise was measured for each incremental curing. The results were tabulated and statistically analyzed by ANOVA test and TUKEY-HSD test.

## RESULTS

It was observed that the temperature rise associated with LED light curing units was considerably lower than QTH units at both 20 and 40 sec exposures from Tables 1 and 2. Among the LED’s at 3 mm distance, the maximum temperature rise of 1.2°C for 20 sec exposure was recorded in Group 2a whereas the least temperature rise of 0.6°C was recorded in Group 1c. The temperature rise associated with Group 3 was also considerably lower at 0.8°C. However, in the QTH units, the maximum temperature rise of 2.0°C was recorded with Group 6, and the least temperature of 1.6°C was recorded with Group 4. Group 5 shows temperature rise of 1.8°C for 20 sec exposure, which is in between the two groups. Thus, the maximum temperature rise of LED units of 20 sec exposure is 1.2°C (Group 2a), whereas the least temperature rise in QTH units is 1.6°C for 20 sec exposure (Group 4). Thus, the LED LC units showed a definite edge over its QTH counterparts.

**Table 1:** Temperature rise in celsius associated with various light curing units at 3mm distance for 20 and 40 sec exposures

Light curing units	20 sec		40 sec		*P* value
	Mean	S.D.	Mean	S.D.	
Group 1-1A	0.80	0.07	1.40	0.16	***
Group 1-1B	1.0	0.100	1.60	0.158	***
Group 1-1C	0.6	0.071	1.20	0.100	***
Group 2-2A	1.20	0.71	1.80	0.71	***
Group 2-2B	1.0	0.071	1.80	0.100	***
Group 2-2C	0.8	0.071	1.40	0.071	***
Group 3	0.8	0.71	1.20	0.71	***
Group 4	1.6	0.158	2.80	0.141	***
Group 5	1.8	0.71	3.00	0.141	***
Group 6	2.0	0.71	3.20	0.158	***

*** *P*<0.001 (statistically significant)

**Table 2:** Temperature rise in celsius associated with various light curing units at 6mm distance for 20 and 40 sec exposures

Light curing units	20 sec		40 sec		*P* value
	Mean	S.D.	Mean	S.D.	
Group 1-1A	0.50	0.071	1.00	0.071	***
Group 1-1B	0.60	0.071	1.20	0.071	***
Group 1-1C	0.40	0.071	0.90	0.071	***
Group 2-2A	0.70	0.100	1.40	0.071	***
Group 2-2B	0.60	0.071	1.20	0.071	***
Group 2-2C	0.50	0.071	1.10	0.071	***
Group 3	0.50	0.71	1.00	0.100	***
Group 4	0.80	0.71	1.40	0.071	***
Group 5	0.90	0.100	1.50	0.071	***
Group 6	1.0	0.071	1.60	0.071	***

*** *P*<0.001 (statistically significant)

At 40 sec exposure also, the temperature increase is associated with exponential rise with all the LED and QTH units. Understandably, here also the LED units fared better than QTH units in causing the least temperature increase.

Considering the various modes of LED LC units, the fast mode caused least thermal emission than the other two modes. Group 1c produced slightly lower temperature rise of 0.6°C than Group 2c of 0.8°C, at 20 sec exposures. In Group 1, the maximum temperature rise of 1.0°C was recorded with ramp mode (Group 1b) for 20 sec exposure, whereas in Group 2 for 20 sec exposure, the maximum temperature rise was observed in Group 2 a of 1.2°C. At 40 sec exposure also, the least temperature rise was associated with Group 1c at 1.2°C whereas maximum temperature rise was seen in both Group 2a and Group 2b at 1.8°C.

Thus, at 3 and 6mm distance for both 20 and 40 sec exposures, the confident LED produced less temperature rise than the Satellac LEDs, but the latter did not lag far too behind. Both the Satellac LEDs caused a little higher temperature than the confident LED and therefore there was no concern of heat damage to the pulp in the case of LED units. However, a QTH unit caused a double fold increase in temperature at both 20 and 40 sec exposures at 3mm distance. Multiple exposures with QTH units can thus cause considerable pulpal damage. When comparing the temperature rise in 3 mm and 6mm distances, 6mm distance produced considerably less temperature rise with all the light curing units in both 20 and 40 sec exposures.

### Temperature rise associated with composite restoration

Temperature rise associated with composite filling was done to simulate the *in-vivo* condition. From Table 3, it is clear that maximum temperature rise was recorded in the cavity preparation prior to placement of bonding agents and composite filling. The temperature rise was observed to decrease after placement of bonding agents, and a slight increase in temperature was recorded after 1^st^ incremental curing. The second and third incremental composite filling produced a lesser degree of temperature rise, thereby indicating the insulating property of composite material. Here again, the LED units produced little temperature increase when compared with QTH units. The least temperature rise was recorded in Group 3, whereas the maximum temperature rise was observed in Group 6. The sum of all the exposures amounted to nearly 5.0°C in both Group 1 and Group 2, whereas it was only 4.0°C in Group 3. However, in QTH unit, the sum of all the exposures amount to above 6.0°C in Group 4 and over 7.5°C in Group 6. Between the three LED’s, Satellac was found to produce the least temperature rise.

**Table 3:** Temperature rise in celsius associated with different light curing units during composite restoration

	After cavity preparation-step-I		After bonding agent application step-II		After 1^st^ incremental filling step -III		After 2^nd^ incremental filling step -IV		After 3^rd^ incremental filling step-V		Post curing step-VI		*P* value
	Mean	S.D.	Mean	S.D.	Mean	S.D.	Mean	S.D.	Mean	S.D.	Mean	S.D.	
Group 1	1.25	0.07	0.55	0.07	0.90	0.00	0.75	0.07	0.55	0.07	0.40	0.00	***
Group 2	1.40	0.00	0.75	0.07	0.95	0.07	0.80	0.00	0.70	0.00	0.50	0.00	***
Group 3	0.90	0.00	0.65	0.07	0.95	0.07	0.70	0.00	0.60	0.00	0.50	0.00	***
Group 4	1.55	0.07	0.90	0.00	1.40	0.00	1.05	0.07	0.80	0.00	0.70	0.00	***
Group 5	1.80	0.00	1.00	0.00	1.45	0.07	1.05	0.07	0.90	0.00	0.80	0.00	***
Group 6	2.00	0.00	1.00	0.00	1.65	0.07	1.25	0.07	1.10	0.00	0.90	0.00	***

*** *P*<0.001 (statistically significant)

## DISCUSSION

Intrapulpal temperature exceeding 42.5°C can result in irreversible damage to the pulp tissue. Polymerization of light-activated resin composites results in a temperature increase caused by both the exothermic reaction process and the energy absorbed during irradiation.[4] Temperature rise increases with increase in radiation time and decrease in material thickness.[8] Masutani *et al*,[10] reported the speed of the exothermic reaction of visible light activated resin composites increases with an increasing intensity of the light source.[10] However, according to Strang *et al*, (1988) the most significant source of heat during the polymerization of a light-activated restorative is from the light activation unit and not from the material itself.

At 6mm distances, the temperature rise was reduced considerably in all the light curing units at 20 and 40 sec exposures which can be attributed to the fact that light intensity rapidly decreases into the depth of the restoration as stated by Kleverlen *et al*.[11] Hence, he recommended curing time up to 40 sec to ensure complete curing of the composites to an adequate depth. Thus, to minimize the effect of heat transfer to the pulp at 6 mm distance, it is always better to use LED LC units for curing.

While evaluating *in vivo* simulation, the sum of all the exposures seems to cause considerable rise in pulpal temperature. The maximum temperature rise was observed at 3.2°C for Cu-100A QTH unit, which is well below the normal range of pulpal physiology. Also, the sum of all the exposures should not be taken into account, since before each exposure a waiting period was observed, which caused the temperature to decrease. When comparing LED units with QTH light curing units, it is always better to use LED unit rather than QTH units, as LED units’ causes minimal thermal emission.

## CONCLUSION

The thermal emission of light curing units per sec and in connection with composite filling can increase pulpal temperature. The following conclusions are drawn from this *in vitro* study:

1. Light emitting diode (LED) light cure units produces significantly less temperature rise than the Quartz-Tungsten-Halogen (QTH) units.
2. Among the various modes in LED units, the Fast mode produces significantly less temperature rise than the Pulse-cure and Ramp modes.
3. At 3 mm and 6 mm distances, the Fast mode of Confident LED light cure units produced less temperature rise, whereas Cu-100A QTH unit produced maximum temperature rise.
4. When five consecutive light curing exposures were applied to the occlusal surface of the prepared teeth, the maximum temperature rise of 2.0°C was measured in the pulp. This could simulate the curing process of a direct inlay or an onlay. When placing a direct composite restoration in three increments, the temperature difference between the start and end of the process was maximally only 3.2°C and thus within the normal range of pulpal physiology.
5. During composite filling, Satellac S.P.LED light cure unit produced the least temperature rise, while Cu-100A QTH unit recorded the maximum temperature rise.
6. The heat emitted by different LED and QTH light curing units varies significantly and it also depends on the curing modes used (in Confident and Satellac Mini LED light cure unit).
